# Three *C. elegans* *srf-6* mutants carry *nsy-1* mutations (*srf-6* is *nsy-1* I)

**DOI:** 10.17912/micropub.biology.000127

**Published:** 2019-07-04

**Authors:** Nicholas D. Van Sciver, Jennifer O. Pulkowski, Samuel M. Politz

**Affiliations:** 1 Department of Biology and Biotechnology, Worcester Polytechnic Institute, Worcester, MA; 2 Present address: McArdle Laboratory for Cancer Research, University of Wisconsin, Madison, WI

**Figure 1.**
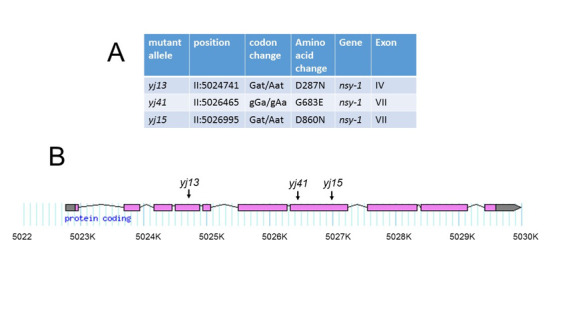
**A.** Table of *srf-6* mutations shown as output of the CloudMap in silico complementation tool. Positions of altered SRF-6 amino acids are numbered as described (Sagasti et al., 2001) **B.** Intron-Exon Map of the *nsy-1* gene showing the position of point mutations originally described as *srf-6* mutations.

## Description

Mutations in the *C. elegans* gene *srf-6* result in an alteration in surface composition detected with monoclonal antibody (mAb), which is specific for the wild-type L1 surface. In *srf-6* mutants, all larval stages display the mAb epitope (Hemmer et al., 1991; Grenache et al., 1996). This suggests that *srf-6* might be a component of a genetic switch that operates in wild-type to turn off expression of the epitope after the L1 stage. We were therefore interested in identifying the *srf-6* DNA sequence.

Genomic DNA was isolated from *srf-6(yj13)*, *srf-6(yj41), and srf-6(yj15)* worms grown on plates, using a Gentra Puregene Kit. Whole genome sequencing of DNA was carried out at the Caltech Genomics Facility. DNA sequences from each mutant were analyzed using the CloudMap workflow (Minevich et al., 2012) on the Galaxy Internet platform (Afgan et al., 2018). After aligning sequences with the *C. elegans* reference genome, variants were called using the GATK Unified Genotyper. To remove variants that were shared in common among all three *srf-6* mutants, the GATK Select Variants plugin was used to subtract the union of two mutant variant sets from the third one. This was repeated for all three pairwise subtractions to produce sets of mutant-specific variants. The effect of each variant on protein structure was predicted, and the resulting sets of predicted changes were merged and subjected to the Cloudmap in silico complementation tool, which sorts variants according to the genetic location and *C. elegans* gene in which they occur.

Results of the *in silico* complementation analysis are shown in Figure 1A. Each strain contained a G to A transition in a chromosome II gene sequence, F59A6.1, which corresponds to the gene *nsy-1.* This result is consistent with the mutations having been induced by ethylmethanesulfonate (Hemmer et al., 1991), and is also consistent with the previous assignment of *srf-6(yj13)* to linkage group II (Grenache et al., 1996).Each mutation resulted in a non-conservative amino acid substitution in the encoded protein, including an aspartic acid changed to asparagine in *srf-6(yj13)*and *srf-6(yj15)*, and a glycine changed to glutamic acid in *srf-6(yj41)*. Positions of these mutations are shown in Figure 1B. Mutations carried by *srf-6(yj15)*and *srf-6(yj41)* were located in exon 7, which contains the kinase catalytic domain (Sagasti et al., 2001). The mutation carried by *srf-6(yj13)* was located in exon 4. All three mutations altered amino acids that are conserved between NSY-1 and its closest human homolog, ASK-1 (Ichijo et al., 1997).

## Reagents

*C. elegans* strains 

AT18 *srf-6(yj13)* II

AT24 *srf-6(yj15)* II

AT25 *srf-6(yj41)* II

Strains will be submitted to the Caenorhabditis Genetics Center.
